# Community‐Based Learning and Its Relevance in Dental Surgeon Education

**DOI:** 10.1111/eje.70163

**Published:** 2026-04-20

**Authors:** Daniela Atili Brandini, Alessandra Marcondes Aranega, Guilherme Assumpção Silva, Vitor Hugo Gonçalves Sampaio, Leonardo Sousa Placco, João Victor de Araújo Narciso, Wilson Galhego Garcia, Cristina Antoniali Silva

**Affiliations:** ^1^ Department of Diagnosis and Surgery São Paulo State University (UNESP), school of Dentistry Araçatuba São Paulo Brazil; ^2^ Department of Basic Sciences São Paulo State University (UNESP), school of Dentistry Araçatuba São Paulo Brazil

**Keywords:** community service‐leaning, dental curriculum, undergraduate dental students

## Abstract

**Background/Objectives:**

Community‐based activities are an essential component of the dentistry curriculum. This study evaluated the perception of undergraduate students participating in Community‐Based Programmes regarding their importance in professional training as dentists.

**Methods:**

Participants of the ‘Happy Smile’ project in 2023 and 2024 were invited via WhatsApp and/or e‐mail to complete a questionnaire based on Likert scales, designed to collect demographic data and assess perceptions related to the development of skills and competencies established by the Brazilian National Curriculum Guidelines for dental education.

**Results:**

Overall, 81.6% of students classified the community‐based learning project as ‘very important’ for their professional training. Among the actions that contributed to the students' educational objectives, the variable ‘importance in student training’ showed positive correlations with participation in organisational and/or feedback meetings (*p* = 0.002), participation in training courses on standard operating protocols (*p* = 0.008), participation in training of educational materials for pedagogical activities (*p* = 0.002), provision of minimally invasive dental care in schools (*p* = 0.002), supervised toothbrushing (*p* = 0.024), and delivery of educational lectures in schools (*p* = 0.006), among others. Key skills and competencies for oral health promotion included the development of non‐verbal communication (*p* = 0.0001), integration into the social context (*p* = 0.022), performance of clinical examination and diagnosis (*p* = 0.0001), adherence to ethical principles (*p* = 0.0001), and teamwork (*p* = 0.0001).

**Conclusions:**

The ‘Happy Smile’ community‐based learning project proved to be highly valuable for the training of future dental professionals across multiple domains. Student involvement in school‐based oral health promotion activities was directly proportional to their self‐confidence and learning outcomes.

## 1 Introduction

Community‐based learning programmes are a fundamental part of the Dentistry curriculum [1]. In community‐based learning activities, undergraduate students have the opportunity to work effectively within the structure and culture of health services, promoting improvements in team performance and enhancing knowledge through theoretical reflection, professional exchange, community experience, and practical work in health facilities [2]. In this context, community‐based programmes are highly relevant within the university and society, as they integrate the community into academic development [3].

For society, Community‐based learning projects play a crucial role in providing free health education, improving health conditions, fostering social change, and consequently enhancing the quality of life of the assisted population [3, 4, 5]. These activities stimulate a transformative process in the thinking of a large portion of underserved communities regarding oral health problems, events often perceived as inevitable or as a natural circumstance associated with their financial condition, such as caries and tooth loss [6].

The principle for training a professional is their interaction with society, which allows integration and identification with the population served, in addition to referencing their technical training with everyday situations [1]. The practise of community‐based learning project strengthens understanding of the health‐disease process and allows students to learn about the economic and social realities of different social scenarios, making them more humanised and capable of promoting health in different communities [1, 4].

In Brazil, in 2018, university community‐based learning was mandatorily integrated into the curriculum of university courses, but it is often still considered a secondary activity. Academic training should train professionals in a scenario of practical experiences to apply the theoretical content received and develop fundamental skills for their professional practise [67]. Thus, community‐based programmes are fundamental for the development of teaching‐learning processes based on everyday life [1, 7]. Such activities promote the complete development of students by improving their communication skills, problem‐solving abilities, and interpersonal skills [7, 8]. This comprehensive teaching is important to meet the objectives proposed by the Brazilian National Curriculum Guidelines (BNCG) for undergraduate dentistry courses, such as health care, decision‐making, communication, leadership, health management, and continuing education [8, 9].

The community‐based projects are characterised by student participation in environments outside the university; democratisation of knowledge through university practises and promotion of participation by the assisted community; integration between teaching and research to meet necessary demands and contribute to the development of social awareness [10, 11].

Thus, these projects are designed to address gaps in oral health prevention and treatment for infants and young children, and they have a major impact on improving overall oral health [2]. Highlighting the importance of these community‐based learning activities in the training of dental surgeons serves as an incentive for the development of new projects and encourages new students to become actively involved in this type of work [11].

By perceiving community‐based programmes as activities capable of providing a more comprehensive academic education and becoming essential elements for student learning, as well as professional and personal development, this study aimed to evaluate the perceptions of undergraduate students who participated in the ‘Happy Smile’ community learning project from November 2023 to November 2024 regarding its importance in their training as dental surgeons.

## 2 Materials and Methods

### 2.1 Ethical Research Procedures

This project was submitted to the Research Ethics Committee (CEP) under Protocol No. 84133524000005420. Prior to the commencement of the study, participants were duly informed about the confidentiality of their identity and the restricted access to personal information through the Free and Informed Consent Form (FICF). The right to voluntarily participate or decline involvement in the research was ensured, as well as the possibility of withdrawal at any stage, without any prejudice or disadvantage. Study participants had the right not to answer any of the questions asked by the researcher if they so wished. Furthermore, the student received comprehensive information regarding the objectives and purposes of the study before consenting to participate.

A copy of the document, containing the researcher's details (DAB), including name, e‐mail address, and telephone number, remained in the possession of the participant, who could contact the researcher at any time during the study to request the deletion of any information that may have been provided until its completion.

### 2.2 Population and Recruitment

Participants were recruited through convenience sampling among undergraduate students enrolled in the Dentistry programme in Araçatuba, who were involved in the ‘Happy Smile’ project between November 2023 and November 2024.

The exclusion criterion encompassed the inability to establish contact with potential participants, as well as the explicit decision not to participate in the study.

### 2.3 Risks and Benefits for the Population Involved in the Research

Participation in this study entailed minimal risks, in accordance with the ethical principles on Research Involving Human Subjects. Participants' identities were not disclosed, and confidentiality was strictly ensured. Accordingly, the data obtained from the questionnaires (responses and transcripts) were stored in a password‐protected file accessible only to the researcher and were transcribed without any identifying information. Regarding benefits, participants contributed both directly and indirectly to the enhancement of public health initiatives, as well as to their own academic training and that of other students.

### 2.4 Material for Evaluating the Perception of Experience

The survey forms (Google Forms) were sent by the authors remotely via communication applications such as WhatsApp or e‐mail.

The students' perception was assessed using a Likert scale, a qualitative, polytomous scale to measure the degree of influence of the ‘Happy Smile’ project on the training of dental students. The questionnaire administered to participants identified demographic data on the population and specific points proposed by the BNCG [8] for dentistry courses, such as: planning health promotion actions, social promotion activities, executing treatment plans, preserving oral health, applying and interpreting scientific research, respecting ethical principles, recognising personal limitations, verbal and non‐verbal communication, teamwork, technological innovation, and continuing education.

Using a Likert scale with the categories *very important, important, moderate, slightly important*, and *not important*, participants were asked how important the ‘Happy Smile’ project was to their training as dental surgeons. In addition, employing a Likert scale with the categories *strongly agree, agree, neutral, disagree*, and *strongly disagree*, participants indicated the extent to which their participation in the project contributed to: 1. Their competencies in performing prevention, promotion, protection, and rehabilitation of oral health; 2. The development of non‐verbal communication; 3. Their competencies in verbal communication; 4. Their competencies in practising the profession in articulation with the social context; 5. Their competencies in planning oral health promotion actions; 6. their competencies in conducting clinical examinations and diagnoses; 7. Their competencies in developing and executing treatment plans; 8. Their competencies in applying and interpreting scientific research on oral diseases and oral health; 9. Their competencies in respecting ethical principles of professional practise; 10. Their competencies in recognising and respecting professional limitations; 11. Their competencies in performing teamwork; 12. Their competencies in monitoring and incorporating technological innovations; 13. Their competencies in participating in continuing education related to oral health and diseases as a component of professional responsibility; 14. Their competencies in exercising leadership skills as dental surgeons; 15. Their competencies in exercising administrative and management skills as dental surgeons; and 16. Their competencies in exercising lifelong learning skills as dental surgeons.

Furthermore, using a numerical scale from 0 to 10, where 0 represented the absence of the need for learning and 10 represented the greatest need for learning, participants were asked to what extent they considered it necessary to improve as dental surgeons in the skills and competencies developed through the *Happy Smile* project. Finally, using a numerical scale from 0 to 10, where 0 represented no importance and 10 represented maximum importance, participants evaluated the significance of specific activities for their professional development, including: 1. Participation in meetings for organisation and feedback on project actions; 2. Participation in the training course on standard operating procedures; 3. Participation in the training course for pedagogical coordinators and presentation of educational materials; 4. Organisation of work schedules; 5. Supervised tooth brushing; 6. Provision of minimal intervention dental care at schools; 7. Data collection for research; 8. Data tabulation for research; 9. Performance of teaching activities; 10. Delivery of educational lectures at schools; and 11. Dissemination of project activities on social media.

### 2.5 Statistical Analyses

Statistical analyses were performed using SPSS version 21.0 (SPSS Inc., Chicago, USA). A significance threshold of α = 0.05 was adopted. Data were structured to evaluate the prevalence of students' opinions according to year of study. Categorical variables were analysed using the Chi‐squared test, followed by Bonferroni post hoc comparisons when appropriate. Continuous variables were examined using one‐way analysis of variance (ANOVA). Correlations among selected variables were assessed using Spearman's rank correlation coefficient.

## 3 Results

Of the 46 students participating in the ‘Happy Smile’ project, 38 undergraduate students from the Araçatuba School of Dentistry—UNESP were included in this study, with a sample power of 90%. There were 14 male students (38.8%) and 24 female students (63.2%). The sample was divided between the second (7.9%), third (18.4%), fourth (15.8%), fifth (50%), and sixth (7.9%) years of the Full‐time, Afternoon‐Evening, and Evening courses.

When questioned regarding the relevance of community‐based programmes in their professional training, the majority of students classified them as ‘very important’ (81.6%) (Table 1). Moreover, a substantial proportion (84.3%) acknowledged that continuous professional development remains necessary to adequately address the demands encountered in daily practise (Table 1).

**TABLE 1:** Students' perception of their need to improve their skills and competencies as dental surgeons (Likert scale from 0 to 10) and the contribution of the ‘Happy Smile’ project to this development. Chi‐squared test with Bonferroni post hoc test for categorical variables and ANOVA for continuous variables (α = 5%).

Student perception							
		Year of enrolment in the undergraduate programme in dentistry					
		2°	3°	4°	5°	6°	Total
How important was the ‘Happy Smile’ project in your training as a dental surgeon? (%)	Very important	100a	71.4a	100a	78.9a	66.7a	81.6
	Important	0a	28.6a	0a	21.1a	0a	15.8
	Median	0a	0a	0a	0a	33.3a	2.6
How much do you think you need to improve in terms of the skills and competencies developed in the project as a dental surgeon? (%)	0–2	0a	0a	0a	0a	0a	0
	3–4	0a	28.4a	0a	15.8a	33.3a	15.8
	5–6	33.3a	14.3a	16.7a	57.9a	33.3a	39.5
	7–8	33.3a	42.9a	50a	15.8a	33.3a	29
	9–10	33.3a	14.3a	33.3a	10.5a	0a	15.8

*Note:* Different letters indicate statistically significant differences between income categories after Bonferroni post‐hoc adjustment, whereas identical letters indicate no significant difference between groups.

The contribution of participation in the ‘Happy Smile’ project activities to oral health promotion, diagnosis, and dental treatment is presented in Table 2. Spearman's correlation analysis indicated that the variables listed in Table 2 did not exhibit statistically significant differences across the years of training (second to sixth).

**TABLE 2:** Students' opinions on the contribution of the ‘Happy Smile’ project to their academic training in oral health promotion, diagnosis, and treatment. Chi‐squared test with Bonferroni post hoc test (α = 5%).

Student perception							
		Year of enrolment in the undergraduate programme in Dentistry					
The project contributes to		2°	3°	4°	5°	6°	Total
Perform oral health prevention and promotion (%)	I totally agree	100a	71.4a	100a	89.5a	66.7a	86.8
	I agree	0a	28.6a	0a	10.5a	33.3a	13.2
Planning of oral health promotion actions (%)	I totally agree	100a	11.4a	100a	68.4a	66.7a	76.3
	I agree	0a	14.3a	0a	31.6a	33.3a	21.1
	Indifferent	0a	14.3a	0a	0a	0a	2.6
Skills to perform clinical examination and diagnosis (%)	I totally agree	100a	85.7a	100a	89.5a	66.7a	89.5
	I agree	0a	14.3a	0a	10.5a	33.3a	10.5
Skills to execute the treatment plan (%)	I totally agree	66.7a	42.9a	83.3a	47.4a	66.7a	55.3
	I agree	33.3a	42.9a	16.7a	47.4	0a	36.8
	Indifferent	0a	14.3a	0a	5.3a	33.3a	7.9
Apply and interpret scientific research on oral diseases (%)	I totally agree	100a	28.6a	83.3a	47.4a	33.3	52.6
	I agree	0a	57.1a	16.7a	26.3a	33.3a	28.9
	Indifferent	0a	14.3a	0a	21.1a	33.3a	15.8
	I disagree	0a	0a	0a	5.3a	0	2.6
Recognise/respect professional limitations (%)	I totally agree	33.3a	57.1a	50a	68.4a	66.7a	60.5
	I agree	33.3a	42.9a	33.3a	26.3a	33.3a	31.6
	Indifferent	0a	0a	16.7a	0a	0a	2.6
	I disagree	33.3a	0a	0a	5.3a	0a	5.3
Respect for ethics in professional practise (%)	I totally agree	66.7a	71.4a	100a	63.2a	66.7a	71.1
	I agree	33.3a	28.6a	0a	36.8a	33.3a	28.9
Monitor and incorporate technological innovations (%)	I totally agree	66.7a	14.3a	50a	21.1a	66.7a	31.6
	I agree	0a	42.9a	33.3a	52.6a	0a	39.5
	Indifferent	0a	42.9a	16.7a	26.3a	0a	23.7
	I disagree	33.3a	0a	0a	0a	33.3a	5.3

*Note:* Different letters indicate statistically significant differences between income categories after Bonferroni post‐hoc adjustment, whereas identical letters indicate no significant difference between groups.

The development of social behaviour, communication, management, and teamwork skills is summarised in Table 3. Although the correlations were not statistically significant, all variables demonstrated a negative association with the years of graduation. This finding suggests that, for students in the early stages of their training, the community‐based learning project provided an important opportunity to develop the competencies outlined in Table 3.

**TABLE 3:** Students' opinions in percentage terms on the contribution of the ‘Happy Smile’ project to their academic training based on practical activities related to behaviour in the social context, administration, and communication. Chi‐squared test with Bonferroni post hoc test (α = 5%).

Student perception							
		Year of enrolment in the undergraduate programme in Dentistry					
The project contributes to		2°	3°	4°	5°	6°	Total
Develop non‐verbal communication (%)	I totally agree	66.7a	57.1a	83.3a	73.7a	66.7a	71.1
	I agree	33.3a	42.9a	0a	15.8a	33.3a	21.1
	Indifferent	0a	0a	16.7a	10.5a	0a	7.9
Verbal communication training (%)	I totally agree	66.7a	71.4a	83.3a	84.2a	100a	81.6
	I agree	33.3a	14.3a	0a	15.8a	0a	13.2
	Indifferent	0a	14.3a	16.7a	0a	0a	5.3
Connection to the social context (%)	I totally agree	66.7a	71.4a	83.3a	78.9a	100a	78.9
	I agree	33.3a	28.6a	16.7a	21.1a	0a	21.1
Perform teamwork (%)	I totally agree	66.7a	71.4a	83.3a	68.4a	66.7a	71.1
	I agree	33.3a	28.6a	16.7a	31.6a	33.3a	28.9
Participate in continuing education in oral health (%)	I totally agree	66.7a	57.1a	100a	73.7a	66.7a	73.7
	I agree	33.3a	42.9a	0a	26.3a	33.3a	26.3
Leadership skills performance (%)	I totally agree	33.3a	42.9a	66.7a	42.1a	66.7a	47.4
	I agree	66.7a	28.6a	0a	36.8a	0a	28.9
	Indifferent	0a	28.6a	33.3a	21.1a	0a	21.1
	I disagree	0a	0a	0a	0a	33.3a	2.6
Training in administration and management skills (%)	I totally agree	66.7a	14.3a	66.7a	42.1a	100a	47.4
	I agree	0a	57.1a	16.7a	26.3a	0a	26.3
	Indifferent	33.3a	26.8a	16.7a	26.3a	0a	23.7
	I disagree	0a	0a	0a	5.3a	0a	2.6
Understanding the importance of continuing education (%)	I totally agree	100a	71.4a	100a	68.4a	66.7a	76.3
	I agree	0a	14.3a	0a	31.6a	33.3a	21.1
	Indifferent	0a	14.3a	0a	0a	0a	2.6

*Note:* Different letters indicate statistically significant differences between income categories after Bonferroni post‐hoc adjustment, whereas identical letters indicate no significant difference between groups.

Students' perceptions regarding the relevance of the project to their acquisition of theoretical knowledge, practical skills, and professional competencies expected of dental surgeons were also assessed (Tables 4 and 5). To better identify which activities of the ‘Happy Smile’ project contributed to students' educational objectives, Spearman's correlation was performed between ‘importance in student education’ (Question 1) and participation in specific activities (Table 4). Significant positive correlations were observed for meetings to organise and/or provide feedback on project actions (*p* = 0.002), participation in the standard operating procedures training course (*p* = 0.008), and participation in the training course for pedagogical coordinators, including the presentation of educational materials for pedagogical activities (*p* = 0.002).

**TABLE 4:** Students' Opinions on the importance of Training Courses for Professional Development. Opinions of undergraduate Dentistry students from the second to sixth years were assessed using a Likert‐type numerical scale, in which 0 indicated ‘no importance’ and 10 represented ‘maximum importance’ of the variable considered. Statistical analysis was performed using ANOVA with Bonferroni post hoc test (α = 5%).

Student perception						
Importance of Training Courses for Professional Development	Year of enrolment in the undergraduate programme in Dentistry					
Mean (SD)	2°	3°	4°	5°	6°	*p*
Participation in meetings for organisation and/or feedback on project actions	7.3 (1.5)	8.4 (1.9)	8.7 (2.2)	8.6 (1.9)	8.7 (2.3)	0.88
Participation in the Standard Operating Procedure course	6 (1.7)	6.4 (3.2)	8.5 (1.9)	8.7 (1.6)	7.7 (2.5)	0.072
Training course for educational coordinators and presentation of educational material	7.7 (2.1)	6.8 (3.4)	8.8 (1.8)	7.4 (2.7)	7.0 (3.0)	0.74

**TABLE 5:** Opinions of undergraduate Dentistry students from the second to sixth years regarding the evolution of their performance after participation in ‘Happy Smile’ project activities were assessed using a Likert‐type numerical scale, in which 0 indicated ‘absence of capacity’ and 10 represented ‘maximum capacity’ for the variable under evaluation. Statistical analysis was performed using ANOVA with Bonferroni post hoc test (α = 5%).

Student perception						
Performance evolution	Year of enrolment in the undergraduate programme in Dentistry					
Mean (SD)	2°	3°	4°	5°	6°	*p*
Work schedule organisation	8.7 (1.5)	8.4 (1.9)	8.7 (2.1)	8.8 (1.7)	9 (1.7)	0.98
Supervised brushing	6.7 (3)	9.6 (1.1)	10 (0)	9.4 (1.3)	9.7 (0.6)	0.016*
Performing minimally invasive dental procedures at school	8.7 (2.3)	7.4 (1.7)	9.8 (0.4)	9.2 (1.6)	9.3 (1.1)	0.06
Data collection	7.7 (2.1)	8.7 (1.4)	9.5 (0.8)	8.6 (1.4)	7.0 (2.6)	0.27
Data tabulation	6.3 (1.5)	8.0 (2.6)	7.3 (2.3)	7.4 (2.6)	6.3 (3.1)	0.84
Performance of teaching activities	6.7 (3.0)	6.3 (3.1)	8.0 (2.3)	7.2 (2.6)	6.7 (4.9)	0.85
Holding educational lectures at the school	7.0 (3.0)	8.8 (2.0)	9.3 (1.6)	8.6 (1.6)	8.0 (1.7)	0.46
Dissemination of project development actions on social media	7.0 (3.0)	7.8 (1.8)	8.5 (1.6)	7.6 (2.8)	8.3 (1.5)	0.89

*Note:* * denotes statistically significant result. P values are for comparisons between groups.

Project activities that demonstrated a significant impact on student training, according to Spearman's correlation test (Table 5), included: organisation of work schedules (*p* = 0.033), minimal intervention dental care at schools (*p* = 0.002), supervised tooth brushing (*p* = 0.024), data collection for surveys and/or research (*p* = 0.031), data tabulation (*p* = 0.012), development of teaching materials for educational activities (*p* = 0.014), educational lectures at schools (*p* = 0.006), and dissemination of project activities on social media (*p* = 0.006).

Furthermore, significant positive correlations were identified between oral health prevention and promotion and the development of several professional skills, including: non‐verbal communication (*p* = 0.0001), articulation in the social context (*p* = 0.022), planning oral health promotion actions (*p* = 0.0001), performing clinical examinations and diagnoses (*p* = 0.0001), executing treatment plans (*p* = 0.001), applying and interpreting scientific research on oral diseases (*p* = 0.007), respecting ethical principles in professional practise (*p* = 0.0001), recognising and respecting professional limitations (*p* = 0.041), teamwork (*p* = 0.0001), monitoring and incorporating technological innovations (*p* = 0.006), participation in continuing education in oral health (*p* = 0.003), and leadership skills (*p* = 0.004).

## 4 Discussion

This study assessed the perceptions of undergraduate Dentistry students regarding the contribution of the Happy Smile community‐based learning programme to their professional training as dental surgeons. A limitation of the study lies in the reflective nature of the questionnaire, which may have prompted participants to formulate responses based on assumptions about their experiences rather than expressing their genuine perceptions. In addition, some responses may have reflected what participants believed the research coordinators expected to read. Another limitation concerns the relative inexperience of students in the early years of their training, which may have influenced the depth and accuracy of their reflections.

Community‐based learning projects are part of the students' educational, cultural, and scientific development, as they articulate teaching, research, and service provision, thereby creating opportunities for the materialisation of the social commitments of universities [3, 12], since they are designed to contribute to the solution of a problem presented by society [12].

University community‐based learning projects offer students the opportunity to improve their reasoning and clinical practise, integrate into the community, and consequently assist their transition into the job market due to direct interaction with patients in different clinical and social situations [13, 14]. This study found that students from all years of training who participate in community‐based learning projects, with a complete and solid structure, have the opportunity to apply newly acquired professional experience, leading them to increased self‐knowledge and maturity [12, 13, 14] in oral health promotion, diagnosis, and dental treatment plans. In this context, the importance of university community‐based learning is evident, as it offers the opportunity to deepen understanding of the interaction between access to health and socioeconomic factors; follow a patient‐centred care model [15]; develop clinical skills based on real experiences; and adapt to the challenges of working in environments with limited resources [16].

Communication, social, and interpersonal skills were also significantly enhanced among most students from the second to sixth years of the dentistry programme who participated in the community‐based learning project. Through the diversification of learning scenarios and methodologies, university community‐based learning activities foster improvements in students' communication and decision‐making abilities by engaging them directly with patients [11, 16, 17]. These activities are also essential for students to practise teamwork and to strengthen their capacity for problem‐solving when confronted with real‐world challenges [18].

Experiences outside the classroom and within the community play a crucial role in the training of future professionals. Enabling them to develop more sensitive and individualised approaches to patient care [19]. Moreover, such experiences promote oral health education and highlight its importance as an integrative vehicle that bridges diverse forms of human knowledge and practise [19, 20]. Training opportunities that expose students to different realities encourage changes in their ways of thinking and acting, ultimately leading to innovative approaches to oral health promotion [2, 4, 6, 21, 22].

Although the digital age does not favour the development of face‐to‐face interpersonal communication or the observation of authentic behaviours, dentistry remains a profession that requires direct and physically close contact with patients. Participation in community‐based learning courses therefore increases the likelihood of students developing these essential skills [23].

Students derive additional value from the community‐based learning experience when guided by a reflective learning process. Enriching such experiences involves photographic documentation, written narratives, and critical incident reports [24]. Furthermore, participants in these experiences should be oriented by principles of partnership, engagement, and capacity strengthening through addressing the demands of the served population [16, 25]. Students enrolled in programmes such as Dentistry seek clinical practises that bring them closer to the realities of the labour market and allow the application of knowledge acquired throughout their training [25, 26]. Studies have demonstrated the importance of learning through social experiences, which foster greater self‐confidence in performing procedures [16]. This was evidenced by the results of the present study, which showed that participation in administrative project meetings and training courses, such as educational lectures in schools and supervised toothbrushing, increased students' perception of their ability to develop various skills and competencies required for promoting oral health [27] in school settings. Supervised toothbrushing was identified as the skill with the lowest self‐confidence performance among second‐year undergraduate students, whereas minimally invasive dental procedures in schools were perceived as having the highest performance among students in the fourth to sixth years of the Dentistry programme.

## 5 Conclusions

The ‘Happy Smile’ community‐based learning project proved to be highly valuable for the training of future dental professionals across multiple domains. Student involvement in school‐based oral health promotion activities was directly proportional to their self‐confidence and learning outcomes.

## Funding

The authors have nothing to report.

## Conflicts of Interest

The authors declare no conflicts of interest.

## Data Availability

Data sharing not applicable to this article as no datasets were generated or analyzed during the current study.
